# Upregulation of Two Cuticular Proteins Is Associated with Resistance to *Beauveria bassiana* in Crowded *Mythimna separata*

**DOI:** 10.3390/insects17040418

**Published:** 2026-04-15

**Authors:** Wenmeng Li, Jing Liao, Lingling Li, Changgeng Dai, Yang Hu, Yuhan Qian, Hongbo Li

**Affiliations:** 1 Key Laboratory of Forest Disaster Warning and Control in Yunnan Province, College of Forestry, Southwest Forestry University, Kunming 650224, China; lwmhbdgh1028@163.com; 2 Guizhou Key Laboratory of Agricultural Biosecurity, Guizhou Branch of State Key Laboratory for Biology of Plant Diseases and Insect Pests, Institute of Plant Protection, Guizhou Academy of Agricultural Sciences, Guiyang 550006, China; liaojing2025@126.com (J.L.); lilingling2019@163.com (L.L.); ggyydai@163.com (C.D.); huyanggz@126.com (Y.H.); 3 Key Laboratory of Crop Genetic Resources and Germplasm Innovation in Karst Region, Ministry of Agriculture and Rural Affairs, Guiyang 550006, China

**Keywords:** *Mythimna separata*, cuticular protein genes, gregarious phase, solitary phase, density-dependent prophylaxis

## Abstract

*Mythimna separata*, an important insect pest of grain crops, exhibits density-dependent prophylaxis (DDP). Most studies have focused on DDP from the perspective of immune system regulation. In this report, we show that two cuticular protein genes, *MsCP1* and *MsCP2,* are involved in DDP of *M. separata*. *MsCP1* and *MsCP2* were highly expressed in 4th and 5th instar larvae of the *M. separata* gregarious high-density phase as compared to solitary, low-density individuals. The expression levels of *MsCP1* and *MsCP2* were significantly higher in the cuticle of gregarious larvae than in solitary individuals. When *MsCP1* and *MsCP2* were knocked down by star polycation (SPc) nanocarrier-mediated RNA interference (RNAi), the expression levels of the two genes were significantly downregulated in gregarious larvae as compared to ds*GFP/SPc*-treated gregarious larvae but were not significantly different from ds*GFP/SPc*-treated solitary larvae. Furthermore, silencing of *MsCP1* and *MsCP2* caused abnormal molting, alterations in ultrastructure, and reduced cuticle thickness in gregarious larvae. The mortality rates in ds*MsCP1/SPc*- and ds*MsCP2/SPc*-treated gregarious larvae were significantly higher after infection with the entomopathogenic fungus *Beauveria bassiana* as compared to the ds*GFP/SPc*-treated gregarious larva but were not significantly different from mortality rates in ds*GFP/SPc*-treated solitary larvae. Taken together, these findings suggest that upregulation of *MsCP1* and *MsCP2* are associated with resistance of gregarious *M. separata* to *B. bassiana*. These results broaden our understanding of insect DDP and are of great significance in the biological control of *M. separata*.

## Simple Summary

*Mythimna separata* exhibits density-dependent prophylaxis (DDP), but mechanisms beyond immune regulation remain underexplored. Here we show that two cuticular protein genes, *MsCP1* and *MsCP2*, contribute to DDP in this pest. Both genes were significantly upregulated in the cuticle of gregarious (high-density) larvae compared to solitary (low-density) individuals. Star polycation (SPc) nanocarrier-mediated knockdown of *MsCP1* and *MsCP2* in gregarious larvae reduced their expression to levels comparable to solitary controls, and led to abnormal molting, reduced cuticle thickness, and altered ultrastructure. Moreover, silenced gregarious larvae showed increased mortality following *Beauveria bassiana* infection, similar to solitary larvae. These results indicate that *MsCP1* and *MsCP2* enhance fungal resistance in crowded *M. separata* by modulating cuticle properties, expanding our understanding of DDP and informing biological control strategies.

## 1. Introduction

Insects represent one of the most successful animal groups in nature, largely because of their highly efficient immune systems that respond to invading pathogens. In addition to innate immunity, insects exhibit plasticity in their immune systems, which allows them to adjust immune capacity according to environmental conditions and physiological states [1,2]. It has been established that changes in population density can alter the immune response of insects [3], and it is generally assumed that high population densities weaken insect immunity due to lack of resources and density-dependent physiological stress [4,5]. Interestingly, some insect taxa exhibit enhanced disease resistance under high population densities, which is termed “density-dependent prophylaxis (DDP)” [6]. The first report of DDP was described in *Pseudaletia separata* [7]; thereafter, this phenomenon was reported in *Tenebrio molitor* [8], *Schistocerca gregaria* [9], *Bombus terrestris* [10], *Locusta migratoria* [11], *Anticarsia gemmatalis* [12], *Plutella xylostella* [13], *Ceratina okinawana* [14] and *Spodoptera litura* [15]. These studies suggest that DDP occurs in a variety of insect species, especially in those that exhibit phase polymorphism.

The underlying mechanisms of DDP have been elucidated in some insect taxa. For example, immune-related parameters such as phenoloxidase activity, lysozyme activity and total hemocyte counts were significantly improved in some species under high-density conditions [9,16,17,18]. Importantly, the induction of DDP can be pathogen-specific in insects. For example, *Mythimna separata* larvae showed the greatest resistance to the bacterial pathogen, *Bacillus thuringiensis*, at a density of 10 larvae/vial, whereas the highest level of resistance to the fungus *Beauveria bassiana* was observed at 30 larvae/vial [18]. This suggests that insects may deploy distinct immune signaling pathways for bacterial and fungal pathogens.

Significant progress has been made in understanding the molecular mechanisms underlying insect DDP in the past decade. Early transcriptomic analyses using RNA-seq revealed that genes encoding protease inhibitors, antioxidants and pattern recognition proteins were upregulated in gregarious locusts as compared to solitary individuals [11]. In recent years, RNA interference (RNAi) technology has emerged as a transformative tool for gene function characterization and pest management [19]. For example, Wang et al. demonstrated that RNA interference (RNAi)-mediated silencing of *gnbp3*, which encodes a pattern recognition protein, significantly improved the susceptibility of gregarious locusts to *Metarhizium anisopliae* [11]. The Toll-Spätzle signaling pathway, a core component of insect innate immune systems, plays a critical role in defense against bacterial, fungal, and other pathogenic infections. In line with this, Jiang et al. reported that the expression of *MsToll-1* and *MsSpätzle-4* in *M. separata* were significantly upregulated with increasing larval density. Moreover, RNAi-mediated knockdown of *MsToll-1* or *MsSpätzle-4* resulted in significant downregulation of multiple immune-related genes in gregarious *M. separata* larvae; more importantly, the survival rate of RNAi-treated gregarious larvae decreased after infection with *B. thuringiensis* [20]. Octopamine, a crucial stress hormone in insects, is involved in various physiological processes, including metabolism, locomotion, and immunity. The octopamine content in the hemolymph of *M. separata* larvae reared under high-density conditions was significantly increased as compared to low-density larvae [18]. Exogenous injection of octopamine significantly enhanced larval phenoloxidase activity and hemocyte counts, whereas injection of its antagonist epinastine suppressed the upregulation of these immune parameters [18]. Furthermore, silencing the gene encoding the octopamine synthesis enzyme, tyramine β-hydroxylase (*Tβh*), in larvae reared in high-density conditions resulted in a 25.1% reduction in octopamine content and a 55.7% decrease in survival of gregarious larvae following exposure to *B. bassiana* [21]. These results suggest that insects perceive signals associated with changes in population density via an unknown mechanism, which subsequently activates the octopamine-mediated neuroendocrine system and Toll–Spätzle immune pathway, ultimately leading to enhanced resistance to pathogens. However, whether other genes or pathways participate in this process is largely unknown.

The insect cuticle is a multilayered structure that functions as an exoskeleton and also provides a physical barrier against adverse environmental factors [22]. The major components of the insect cuticle are cuticular proteins (CPs), chitin, lipids and water [23]. It is well-established that cuticular proteins play an important role in insect growth and development [24,25,26,27]. Interestingly, recent studies have shown that CPs are involved in insect resistance to pesticides [28]. Long-term insecticide exposure improves cuticle thickness and upregulates the expression of genes encoding CPs, thereby decreasing the ability of insecticides to traverse the cuticular barrier [29,30,31,32,33]. Similarly, entomopathogenic fungi adhere to and grow on the insect exoskeleton, ultimately penetrating the cuticle and moving into hemolymph. It is not clear whether cuticular proteins participate in insect DDP.

The oriental armyworm, *Mythimna separata*, is an important pest of grain crops in Asia and exhibits DDP [34]. Our recent studies demonstrated that gregarious, high-density larvae of *M. separata* have thicker cuticles and greater expression of cuticular proteins than solitary, low-density larvae [35], leading us to hypothesize that cuticular proteins are critical for DDP. To test this hypothesis, a reliable gene function analysis tool is required. RNAi has been widely used for studying gene functions in insects; however, its efficiency is often low in lepidopteran species due to the existence of dsRNA-degrading nucleases in the hemolymph and midgut [36]. To overcome these barriers, various nanomaterial-based dsRNA delivery systems have been developed, including chitosan nanoparticles, liposomes, carbon nanotubes, layered double hydroxides, and star polycation (SPc) [37,38,39]. Among these, SPc-mediated dsRNA delivery has been successfully applied in multiple insect species, significantly improving RNAi efficiency compared with naked dsRNA [40,41,42,43]. In the present study, we first identified two genes encoding cuticular proteins (designated *MsCP1* and *MsCP2*) from the *M. separata* transcriptome database [34] and analyzed their transcription in different developmental stages and tissues of solitary and gregarious larvae. Subsequently, we employed SPc-mediated RNAi targeting *MsCP1* and *MsCP2* to evaluate their impact on cuticle morphology and resistance to *B. bassiana*. Our study provides insight into the role of *M. separata* cuticular proteins in DDP and further broadens our understanding of this phenomenon, thus revealing potential novel management strategies for this insect pest.

## 2. Materials and Methods

### 2.1. Insect Colonies and Fungal Strain

The long-term laboratory population of *M. separata* used in this study was originally collected as larvae on maize in Qianxi City, China, in 2014. The larval colony containing both solitary and gregarious *M. separata* was established as described previously [44] and reared on a corn-based diet at 25 ± 1 °C with a 14:10 h light/dark photoperiod. Emerging adults of both phases were transferred to nylon cages and provided with a 10% honey/water diet and plastic ropes for oviposition.

The star polycation (SPc) nanocarrier used to delive dsRNA was provided by professor Yan at China Agricultural University and stored at 4 °C. The *Beauveria bassiana* strain used in this study was obtained from Shanxi Luhai Pesticide Technology Co., Ltd. (Shanxi, China) and stored at −80 °C until needed. Prior to experiment, the *B. bassiana* strain was inoculated onto potato dextrose agar (PDA) medium (potato 200 g, glucose 20 g, agar 20 g, peptone 5 g, distilled water 1000 mL) and cultured in an artificial climate chamber at 26 °C ± 1 °C, relative humidity 80% ± 5%, and a photoperiod of 12 h light: 12 h dark for 10 days. Based on the preliminary experiment, the *B. bassiana* spore was diluted with DEPC-treated water to a concentration of 1.0 × 10^7^ CFU/mL.

### 2.2. Bioinformatic Analysis of MsCP1 and MsCP2

The cDNA sequences of *MsCP1* and *MsCP2* were obtained from our recently sequenced *M. separata* transcriptome database [43]. Nucleotide and deduced amino acid sequences of *MsCP1* and *MsCP2* were identified with ORF finder (https://www.ncbi.nlm.nih.gov/orffinder/), and SMART (http://smart.embl.de/) was used to predict conserved domains. Deduced CP amino acid sequences from other insects were compared with MsCPs using DNAMAN (https://www.lynnon.com/), and a phylogenetic tree was constructed using MEGA v. 7.0 and the neighbor-joining algorithm with 2000 repetitions.

### 2.3. Expression Patterns of MsCP Genes in Gregarious and Solitary M. separata

Samples were collected from 4th to 6th instar larvae because the crowding effect becomes apparent only after the 3rd instar stage. The tissues including heads, cuticles, hemolymph, fat bodies, guts and feet were collected from the 5th instar larvae of both phases. Total RNA of each sample was extracted using Eastep^®^ Super Total RNA Isolation Kit following the manufacturer’s protocol (Promega, Madison, WI, USA), and genomic DNA was removed with DNase I. After RNA quality was checked and concentrations were determined, the iScript cDNA Synthesis Kit (Bio-Rad, Hercules, CA, USA) was used to synthesize first-strand cDNA from total RNA. Quantitative real-time PCR (qRT-PCR) was performed using a CFX96 Real-time PCR System (Bio-Rad, Hercules, CA, USA) as follows: 95 °C for 2 min, followed by 35 cycles of 95 °C for 5 s, and 30 s at 60 °C. Melting curves for each primer pair were determined, and β-Actin and Tubulin were used as reference genes for developmental stages and tissues, respectively [45]. The 2^−ΔΔCt^ method was utilized to determine expression levels of the two *MsCPs*. Experiments consisted of three independent biological replications, with each replicate containing 3–5 larvae. Primers used in the experiment are listed in Table S1.

### 2.4. RNAi of MsCP1 and MsCP2

The double-stranded RNAs (dsRNAs) for *MsCP1* and *MsCP2* were amplified using gene-specific primers with integrated T7 promoters (Table S1). dsRNA synthesis was performed using the Transcript Aid T7 High Yield Transcription Kit (Thermo Scientific, Waltham, MA, USA). The synthesized dsRNA was purified and dissolved in ddH_2_O at 5 µg/µL. Owing to the low efficacy of RNAi in lepidopteran species, a star polycation (SPC) nanocarrier was used to deliver the dsRNA according to the previously described method with minor modifications [40]. Briefly, the dsRNA samples and SPc were diluted in DEPC-treated water to concentration of 5 μg/μL and 10 μg/μL, respectively. Then, two volumes of the dsRNA and one volume of SPc solution were mixed thoroughly and incubated at 25 °C for 30 min to allow for the formation of dsRNA/SPc complexes. Finally, this solution was diluted with DEPC-treated water to a final concentration of 2.5 μg/μL. On the first day of the 4th instar larval stage, individuals from gregarious (G) and solitary (S) phases were used in RNAi experiments. Each larvae was injected with 1 μL ds*RNA/SPc* (2.5 µg/μL). Treatments included the following: (1) solitary larvae injected with dsGFP/SPc (S-ds*GFP/SPc*); (2) gregarious larvae injected with ds*MsCP1/SPc* (G-ds*MsCP1/SPc*); (3) gregarious larvae injected with ds*MsCP2/SPc* (G-ds*MsCP2/SPc*); and (4) gregarious larvae injected with ds*GFP/SPc* (G-ds*GFP/SPc*). At 48 h after injection, samples were collected for RNA extraction, and qRT-PCR was conducted to determine the interference efficiency of *MsCP1* and *MsCP2* expression as described above. Phenotypes for each treatment were observed and photographed with a digital camera (DS-Fi3, Nikon, Tokyo, Japan). Each treatment observed 20 larvae.

### 2.5. Scanning Electron Microscopy

To evaluate the potential effects of *MsCPs* knockdown on cuticle development, the ultrastructure of larval cuticles from the above four treatments were analyzed by scanning electron microscopy (SEM). Cuticles on the dorsal side of solitary and gregarious larvae were dissected at 72 h after RNAi. Dissected samples were washed with PBS (pH 7.4), fixed for 2 h at room temperature, and stored at 4 °C until needed. Fixed samples were then washed three times in 0.1 M PBS for 15 min. Samples were transferred to 1% O_s_O_4_ for 2 h and incubated at room temperature in darkness, followed by three washes with 0.1 M PBS as described above. Washed cuticles were then incubated for 15 min in a graded ethanol series (30, 50, 70, 80, 90, 95 and 100%) and then dried in an EM CPD300 critical point dryer (Leica, Solms, Germany). Samples were then coated with gold for 30 s using a K550X sputter coater (Electron Microscopy Sciences, Hatfield, PA, USA). Finally, a TM-1000 SEM (Hitachi, Tokyo, Japan) was used to record images at 3 kV. Since we did not observe any differences in cuticles of the same phase [35], the middle regions of cuticles were compared. Five individuals from each phase were used for SEM analysis.

### 2.6. Transmission Electron Microscopy

Cuticle thickness in solitary and gregarious larvae treated with dsRNAs was evaluated by transmission electron microscopy (TEM). Cuticles from the four treatments (Section 2.4) were prepared for TEM as described previously [29], and a JEM-1210 transmission electron microscope (HT7800, Hitachi, Tokyo, Japan) was used to observe dissected cuticles at 80 kV. Digital images for each sample were photographed and used to measure thickness with Image J2 (https://imagej.net/ij/). Since no obvious differences were apparent among different cuticular sections in our previous study [34], five cuticular regions were randomly selected in gregarious (n = 4) and solitary larvae (n = 4) and analyzed for thickness.

### 2.7. Resistance to B. bassiana

To evaluate the effects of *MsCP1* and *MsCP2* knockdown on disease resistance, surviving larvae were collected at 24 h after injection and exposed to a *B. bassiana* suspension containing 1.0 × 10^7^ CFU/mL. Larvae were exposed to the *B. bassiana* solution for 5 s, air-dried at room temperature and transferred to their initial rearing conditions. Survival rates were recorded daily until larvae either died or pupated. This experiment included three independent biological replications, with each replicate containing 20 larvae.

### 2.8. Statistical Analyses

Significant differences among treatments were determined by one-way ANOVA, followed by Tukey’s honest significant difference (HSD) test. Student’s *t*-test was used to assess differences between the gregarious and solitary larval phases, and significance at *p* < 0.05 (*), *p* ≤ 0.01 (**), and *p* ≤ 0.001 (***) was determined. Datapoints represent as means ± standard error (SE). All statistical analyses were carried out using DPS v. 17.0 software [46].

## 3. Results

### 3.1. Bioinformatic Analysis of MsCP1 and MsCP2

Two cuticular protein genes were identified in the *M. separata* transcriptome and designated *MsCP1* and *MsCP2*; these two genes encoded 396 and 336 bp ORFs with deduced protein sequences of 131 and 111 amino acids, respectively (Figure S1). Sequence analysis showed that both CPs contained a chitin-binding domain in the RR1 subfamily. The two MsCPs and CPs from other insect species were analyzed for phylogenetic relatedness. As shown in Figure S2, the phylogenetic tree was clearly divided into two branches, including Lepidoptera species and other species. Within Lepidoptera branch, MsCP1 and MsCP2 clustered with CP in *Mythimna loreyi* on a branch adjacent to CP in Colics croceus.

### 3.2. Expression of MsCP1 and MsCP2 in Gregarious and Solitary M. separata

**Figure 1:**
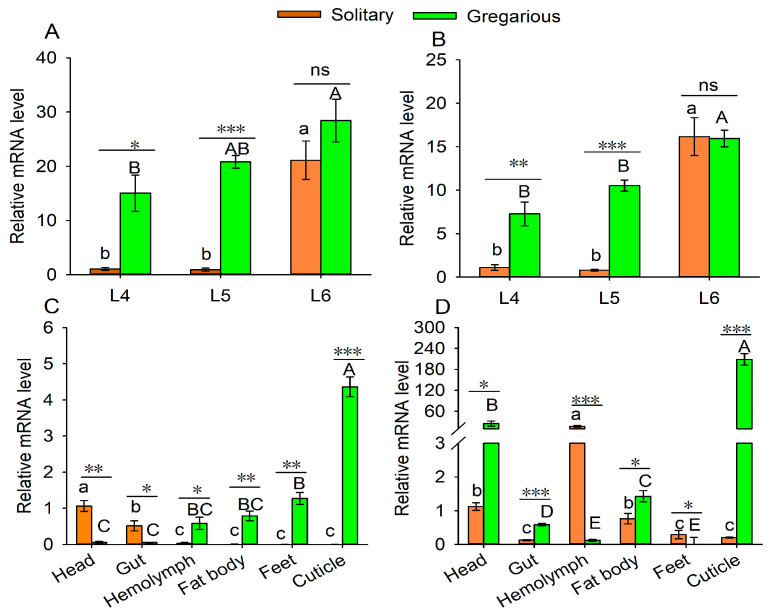
Expression levels of *MsCP1* and *MsCP2* in gregarious and solitary *M. separata*. Panels (**A**,**B**) show expression levels of *MsCP1* and *MsCP2* across developmental stages of gregarious and solitary *M. separata*, respectively. Panels (**C**,**D**) illustrate tissue-specific expression patterns of *MsCP1* and *MsCP2* in 5th instar larvae of gregarious and solitary *M. separata*. Abbreviations: L4–L6, the fourth- to sixth- instar larvae. Data represent means ± SE (n = 3). Different lowercase or capital letters indicate significant differences among stages or tissues at *p* ≤ 0.05 based on one-way ANOVA with Tukey’s honest significant difference test. Asterisks (*), (**) and (***) indicate significant differences among gregarious and solitary larvae at *p* ≤ 0.05, *p* ≤ 0.01 and *p* ≤ 0.001, respectively. The notation “ns” indicates no significant difference between the two phases at *p* ≤ 0.05.

The developmental and tissue-specific expression patterns of the two *MsCPs* were analyzed by qRT-PCR. As shown in Figure 1, the two CP genes were differently expressed in 4–6th instar larvae and were up-regulated as development proceeded (*MsCP1*: *F_solitary_* = 31.96, *p* = 0.0006; *F_gregarious_* = 4.83, *p* = 0.0362; *MsCP2*: *F*_solitary_ = 47.642, *p* = 0.0002; *F*_gregarious_ = 17.921, *p* = 0.0029). When subjected to *t*-test analysis, the expression levels of *MsCP1* and *MsCP2* were higher in gregarious 4th and 5th instar larvae as compared to solitary larvae, but there was no significant difference between the two phases when analyzed for expression in 6th instar larvae (Figure 1A,B).

Tissue distribution analysis showed that the two CP genes were differentially expressed in the various tissue types (*MsCP1*: *F_solitary_* = 27.66, *p* < 0.001; *F_gregarious_* = 105.27, *p* < 0.001; *MsCP2*: *F_solitary_* = 31.56, *p* < 0.001; *F_gregarious_* = 122.40, *p* < 0.001). For example, *MsCP1* expression was higher in the heads and midguts of solitary individuals as compared to insects in the gregarious phase; however, expression levels in the hemolymph, fat bodies, foot, and cuticle were higher in gregarious larvae than in solitary individuals (Figure 1C,D). *MsCP2* expression was lower in the hemolymph and feet of gregarious vs. solitary larvae but was more highly expressed in heads, midguts, fat bodies, and cuticles of gregarious larvae as compared to solitary individuals (Figure 1C,D).

### 3.3. Knockdown of MsCP1 and MsCP2 Expression by RNAi

**Figure 2:**
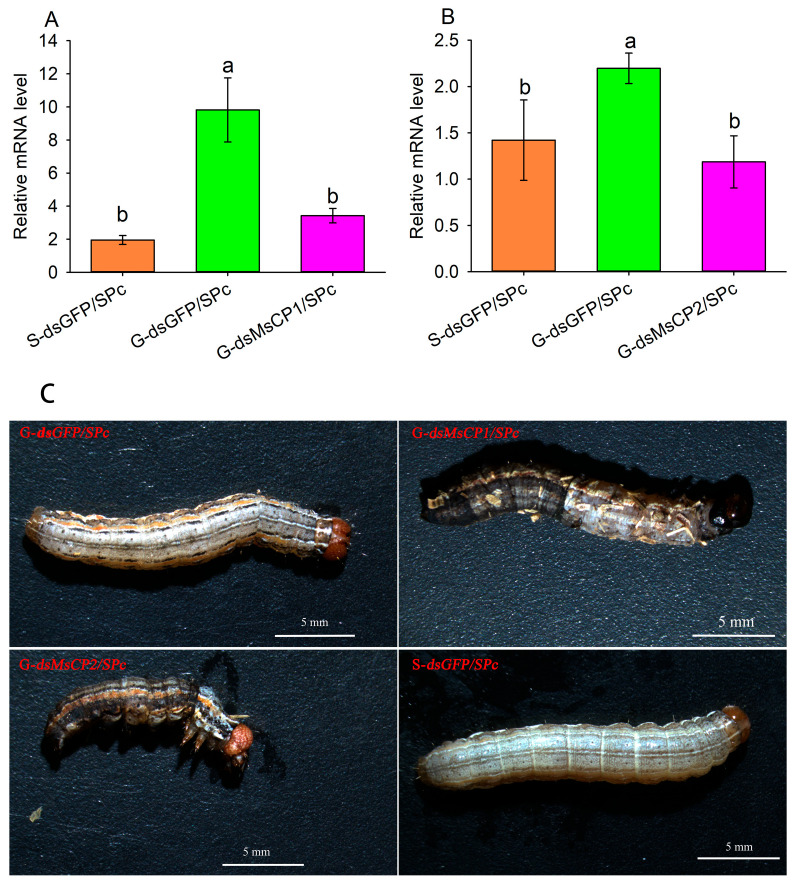
Silencing efficiency of *MsCPs* and larval phenotypes after SPc-mediated RNAi. Panels (**A**,**B**) show expression levels of *MsCP1* and *MsCP2* at 48 h after injection with dsRNA*/SPc*, respectively. Panels (**C**) show phenotypes of solitary and gregarious larvae injected *dsRNA/SPc*. Abbreviations: S-ds*GFP/SPc*, solitary larvae injected with ds*GFP/SPc*; G-ds*GFP/SPc*, gregarious larvae injected with ds*GFP/SPc*; G-ds*MsCP1/SPc* and G-ds*MsCP2/SPc*, gregarious larvae injected with ds*MsCP1/SPc* and ds*MsCP2/SPc*, respectively. *β*-actin was used as a reference gene. Data points represent means ± SE from three independent biological replications and three technical replications. Different lowercase letters indicate significant differences among treatments at *p* ≤ 0.05 based on one-way ANOVA with Tukey’s honest significant difference test.

To explore the function of *MsCP1* and *MsCP2*, we silenced the two genes by injecting ds*CP/SPc complexes* into 4th instar larvae of gregarious and solitary *M. separata*. The expression levels of *MsCP1* and *MsCP2* in gregarious larvae treated with ds*MsCP1/SPc* or ds*MsCP2/SPc*, respectively, were 65.12% and 46.00% lower compared than the control (G-ds*GFP/SPc*) and were not significantly different from expression in solitary larvae treated with ds*GFP* (S-ds*GFP/SPc*, Figure 2A,B). Knockdown of *MsCP1* and *MsCP2* expression resulted in abnormal molting in gregarious larvae as compared to solitary and gregarious larvae treated with ds*GFP/SPc* (Figure 2C).

### 3.4. SEM Analysis of Cuticles After Silencing MsCPs Expression

**Figure 3:**
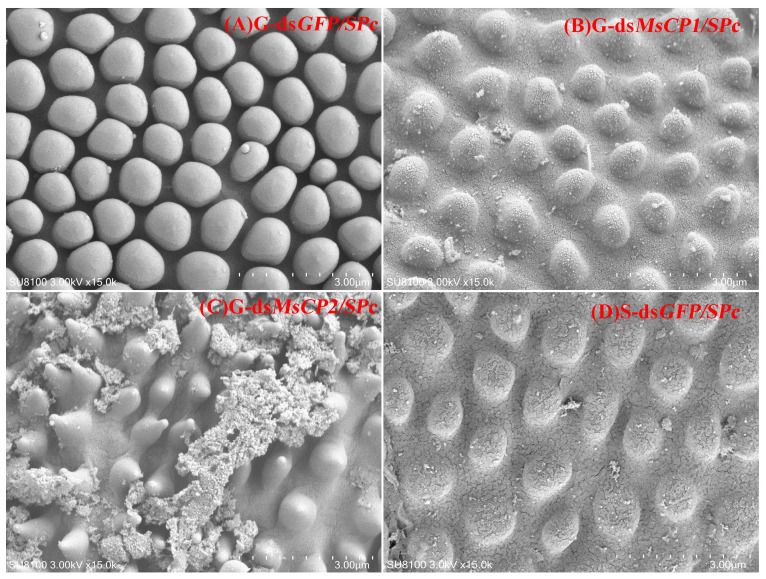
SPc-mediated RNAi of *MsCP1* and *MsCP2* changes cuticle ultrastructure of gregarious larvae. Panels (**A**,**D**) represent cuticle ultrastructure in gregarious (G) and solitary (S) larvae injected with ds*GFP/SPc*, respectively. Panels (**B**,**C**) show cuticle ultrastructure in gregarious larvae injected with ds*MsCP1/SPc* or ds*MsCP2/SPc*, respectively. The image was generated with a TM-1000 scanning electron microscope at a 3-kV acceleration voltage.

To further examine the roles of *MsCP1* and *MsCP2*, SEM was used to observe cuticle ultrastructure in both gregarious and solitary larvae injected with ds*MsCP1/SPc*, ds*MsCP2/SPc* or ds*GFP/SPc* (Figure 3). Results showed that silencing *MsCP1* or *MsCP2* in gregarious larvae resulted in blurry cuticular surfaces (Figure 3B, ds*MsCP1/SPc*) or upward-facing polygonal particles (Figure 3C, ds*MsCP2/SPc*) when compared to ds*GFP/SPc*-treated gregarious larvae (Figure 3A, G-ds*GFP/SPc*). Furthermore, the distance between particles in ds*MsCP1/SPc* and ds*MsCP2/SPc*-treated gregarious individuals were larger, which was similar to results observed in dsGFP*/SPc*-treated solitary larvae (Figure 3D, S-ds*GFP/SPc*).

### 3.5. TEM Analysis of Cuticle Thickness After MsCPs Knockdown

**Figure 4:**
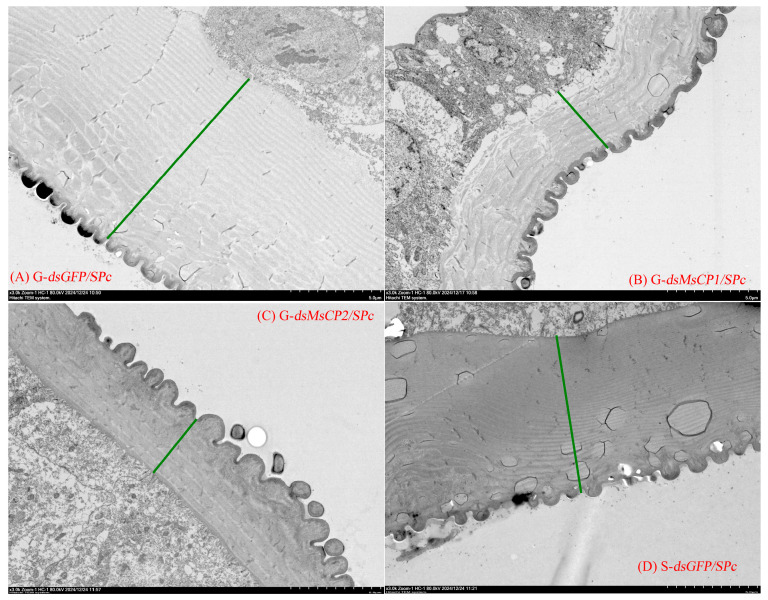
Transmission electron microscopy demonstrated that knockdown of *MsCP1* and *MsCP2* via SPc-mediated RNAi modifies cuticular morphology and thickness in gregarious larvae of *M. separata*. Panels (**A**,**D**) represent TEM images of longitudinal sections of cuticles from gregarious (G) and solitary (S) larvae injected with ds*GFP/SPc*, respectively. Panels (**B**,**C**) show TEM images of longitudinal cuticular sections in gregarious larvae injected with ds*MsCP1/SPc* and ds*MsCP2/SPc*, respectively. Images were obtained with a JEM-1210 transmission electron microscope at 80 kV. Green lines illustrate cuticle thickness in gregarious and solitary larvae.

**Figure 5:**
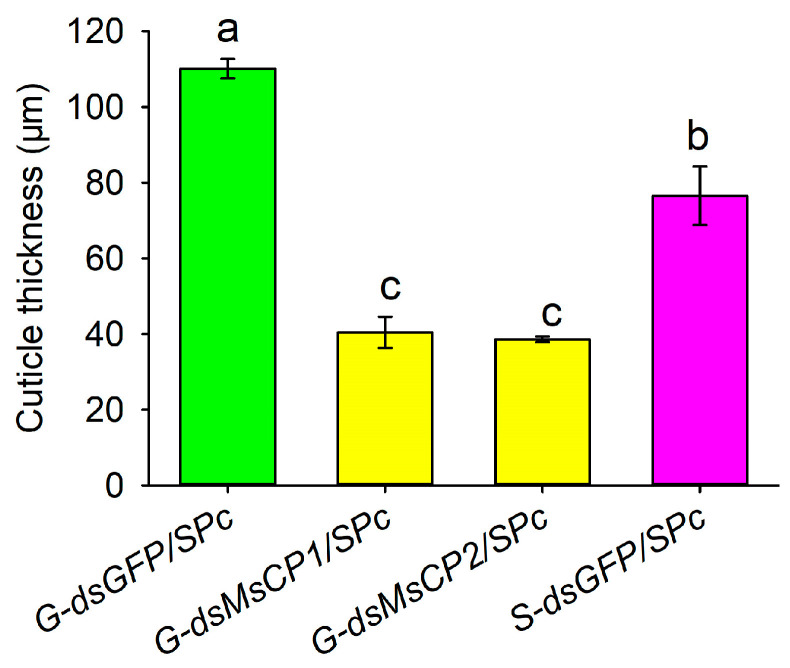
SPc-mediated RNAi of *MsCP1* and *MsCP2* decreases cuticle thickness of gregarious larvae. Abbreviations: S-ds*GFP/SPc*, solitary larvae injected with ds*GFP/SPc*; G-dsGFP*/SPc,* gregarious larvae injected with dsGFP*/SPc*; G-ds*MsCP1/SPc* and G-ds*MsCP2/SPc*, gregarious larvae injected with ds*MsCP1/SPc* and ds*MsCP2/SPc*, respectively. Data represent means ± SE from four independent biological replications. Different lower letters indicate significant differences among treatments at *p* ≤ 0.05 as calculated by one-way ANOVA with Tukey’s honest significant difference test.

Longitudinal sections of cuticles were relatively smooth in ds*GFP/SPc*-treated larvae, whereas the cuticular surface became more uneven and exhibited fissures in ds*MsCP1/SPc*- and ds*MsCP1/SPc*-treated larvae (Figure 4). Furthermore, cuticle thickness in ds*MsCP1/SPc*- or ds*MsCP2/SPc*-treated gregarious larvae was significantly lower (ds*MsCP1/SPc*: 40.42 µm, ds*MsCP2/SPc*: 38.54 µm) as compared to ds*GFP/SPc*-treated gregarious larvae (110.12 µm) and was also less than thickness in ds*GFP/SPc*-treated solitary larvae (72.37 µm) (Figure 4 and Figure 5).

### 3.6. Assays for Resistance to B. bassiana

**Figure 6:**
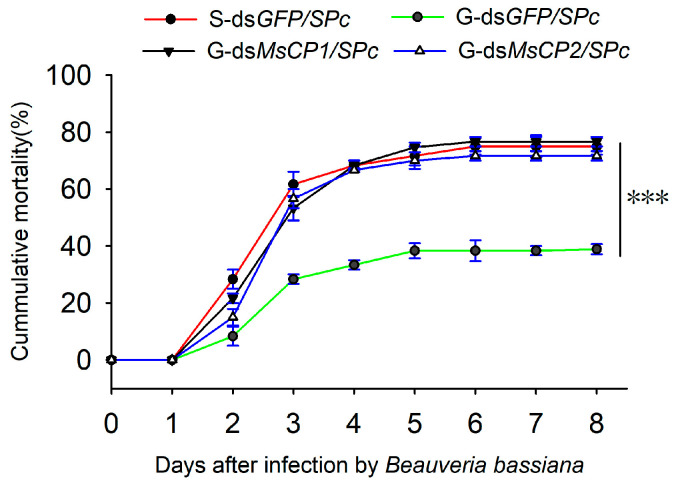
SPc-mediated RNAi of *MsCP1* and *MsCP2* increases the mortality of gregarious *M. separata* larvae infected with *B. bassiana*. Abbreviations: S-ds*GFP/SPc*, solitary larvae injected with ds*GFP/SPc*; G-dsGFP*/SPc,* gregarious larvae injected with dsGFP*/SPc*; G-ds*MsCP1/SPc* and G-ds*MsCP2/SPc*, gregarious larvae injected with ds*MsCP1/SPc* and ds*MsCP2/SPc*, respectively. Asterisks (***) indicate significant differences between the two phases of *M. separata* at *p* ≤ 0.001 as calculated by Student’s *t*-test.

As shown in Figure 6, the mortality of both ds*GFP/SPc*-treated solitary and gregarious larvae gradually increased after exposure to *B. bassiana*. The cumulative mortality of ds*GFP/SPc*-treated gregarious larvae (38.33%) was significantly lower than ds*GFP/SPc*-treated solitary larvae (75.00%) at the 8th d after infection with *B. bassiana*. However, the cumulative mortality rates in ds*MsCP1/SPc*- or ds*MsCP2/SPc*-treated gregarious larvae (ds*MsCP1/SPc*: 76.67%; ds*MsCP1/SPc*: 71.66%) increased after *B. bassiana* infection as compared to the ds*GFP/SPc*-treated gregarious control (G-ds*GFP/SPc*).

## 4. Discussion

A major factor contributing to substantial yield losses in crops infected with *M. separata* is the formation of the gregarious phase in response to high population density. Notably, gregarious *M. separata* larvae exhibit improved immunity and decreased susceptibility to fungal biopesticides, thereby facilitating outbreaks in field crops [47]. In a prior study, we showed that gregarious *M. separata* larvae are more resistant to *B. bassiana* than their solitary counterparts [34]. Nevertheless, the mechanistic basis of DDP in *M. separata* remains unclear. Several studies have suggested that neurohormones and the Toll–Spätzle immune pathway provide a more effective defense against pathogens in *M. separata* [18,20,21]. In the current study, we identified two cuticular protein genes, *MsCP1* and *MsCP2*, which improved the resistance of gregarious *M. separata* to *B. bassiana* by modifying cuticle ultrastructure and thickness. To our knowledge, this is the first report providing insight into the role of cuticular proteins in insect DDP.

To explore the biological function of cuticular proteins in DDP, we first examined the expression of *MsCP1* and *MsCP2* in the two phases of *M. separata*. Expression of these genes was higher in 4th and 5th instars of gregarious *M. separata* larvae than in solitary individuals. This result was similar to those reported in previous studies, where an obvious upregulation of stress-related genes was observed in both gregarious *M. separata* and locusts [44,48,49]. The upregulation of the two *MsCPs* is presumably due to the crowding that occurs in the 4th and 5th larval stages of *M. separata*. In addition, the elevated expression of these cuticular protein genes suggests that *M. separata* possibly reinforces its cuticle as a defensive strategy.

In this study, SPc-mediated RNAi targeting *MsCP1* and *MsCP2* were performed in 4th instar larvae of gregarious *M. separata*. Injection with ds*MsCP1/SPc* and ds*MsCP2/SPc* altered the morphology of gregarious larvae and resulted in a smaller body size and abnormal molting, suggesting a role for *MsCP*s in cuticle development. The altered larval morphology induced by ds*MsCP1/SPc* and ds*MsCP2/SPc* treatment were further investigated by SEM. Consistent with previous findings [35], ds*GFP/SPc*-treated gregarious larvae exhibited more polygonal particles on the cuticular surface and showed higher resistance to *B. bassiana* than ds*GFP/SPc*-treated solitary larvae. Similarly, Grizanova et al. reported that melanic *G. mellonella* larvae had a greater number of melanized spots on the cuticle surface as compared to non-melanic morphs, and melanic larvae had a higher level of resistance to entomopathogenic fungi than non-melanic counterparts [50]. The dense polygonal particles on the cuticular surface of gregarious larvae may physically reduce the contact area available for conidial attachment or alter surface free energy and wettability, thereby decreasing conidial adhesion efficiency. This speculation was supported by previous studies showing fewer conidial adhered to and germinated on the cuticle of insects exhibiting resistance to fungal pathogens [50,51,52]. In other words, the enhanced resistance of gregarious larvae may primarily depend on a physical “barrier effect” of the cuticular surface architecture rather than solely on immune activation. As noted in the current study, SPc-mediated RNAi targeting *MsCP1* in gregarious larvae resulted in blurred cuticular edges, whereas SPc-mediated RNAi targeting *MsCP2* resulted in upward-facing polygonal particles. Both alterations likely disrupt the original smooth or compact surface topology, providing more favorable attachment sites for conidial. Consistent with this, mortality rates of ds*MsCP1/SPc*- and ds*MsCP2/SPc*-treated gregarious larvae exposed to *B. bassiana* were significantly higher than those of ds*GFP/SPc*-treated gregarious individuals. These results support the mechanistic inference that specific cuticular ultrastructure regulates antifungal resistance by influencing conidial attachment efficiency. Our study was similar to a previous study in *Tribolium castaneum* [53], in which combined knockdown of adult-specific cuticular protein genes weakened antifungal phenotypes, improved germination of conidial attached on the adult body surface, and increased the susceptibility of host to two fungi biopesticides. Taken together, we propose that *MsCP1* and *MsCP2* contribute to the construction of a specific cuticular surface ultrastructure that indirectly modulates conidial attachment and germination in gerearious larvae. Although the infection process of conidial on the cuticle was not directly observed, the above reasoning suggests that changes in cuticular ultrastructure at least partially explain the enhanced resistance of gregarious larvae to *B. bassiana*. Given that the cuticle is primarily composed of structural cuticular proteins, chitin, and lipids [23], with the latter two components also serving important protective functions [54,55], we further speculate that silence of the two *MsCPs* may alter the arrangement or cross-linking status of cuticular components, thereby affecting the physicochemical properties of cuticle constituents [55] and ultimately weakening antifungal defense of gregarious larvae. Nevertheless, further studies are needed to validate this speculation.

Another notable phenotype was that ds*GFP/SPc*-treated gregarious larvae exhibited thicker cuticles than ds*GFP/SPc*-treated solitary larvae. SPc-mediated silence of *MsCP1* and *MsCP2* decreased cuticle thickness in gregarious larvae and resulted in high mortality following exposure to *B. bassiana*. These data suggest that the thicker cuticle of gregarious larvae acts as a physical barrier against fungal hyphal penetration. Mechanistically, increased cuticle thickness primarily extends the distance that fungal hyphae must traverse and increases the total amount of cuticular matrix that must be degraded. Because entomopathogenic fungi must secrete proteases and chitinases to degrade the cuticle before reaching the hemolymph, a thicker cuticle requires more time and greater enzymatic investment for successful penetration. The phenomenon of cuticle thickening mediating resistance to chemical insecticides has been well documented in several insect species, often involving in overexpression of CP genes [28]. For example, when the CP-encoding gene *SlMsCP26* was silenced, cuticle thickness decreased, which caused increased susceptibility to indoxacarb in resistant strains of *Spodoptera litura* [32]. In mosquitos, knockdown of multiple CP genes resulted in thinner legs and resulted in higher mortality after insecticide exposure [29,30,31,32]. RNAi-mediated silencing of the CP-encoding gene, *BgCPLCP1*, resulted in a thinner cuticle and higher mortality to the insecticide β-cypermethrin in cockroaches [56]. Although these studies focused on chemical insecticides, the physical barrier logic is comparable to fungal penetration: chemical insecticides enter primarily through cuticular penetration, while fungi must physically breach the cuticle. Therefore, we propose that gregarious larvae, through MsCPs-mediated cuticle thickening, increase the time and energy costs required for fungal hyphae to penetrate the cuticle, thereby delaying or reducing the probability of successful fungal entry into the hemolymph. Given that insect mortality depends on fungal ingress into the hemolymph [57], this thickness-dependent “penetration retardation mechanism” reasonably explains the differential susceptibility to *B. bassiana* between gregarious and solitary larvae. Furthermore, this retardation mechanism may provide a broader time window for the activation of humoral immune responses (e.g., the phenoloxidase system and antimicrobial peptide synthesis), thereby creating a synergistic effect between physical barrier and immune defense and resulting in a stronger resistance phenotype.

It has been demonstrated that efficient knockdown of *MsCP1* and *MsCP2* can be achieved by injecting dsRNA/SPc complexes. Although injection enables precise delivery of dsRNA doses and rapid systemic distribution, this method is labor-intensive and impractical for large-scale pest management. From a practical standpoint, oral delivery (i.e., feeding dsRNA/SPc to larvae) would be far more feasible for potential field applications. Notably, a recent study has shown that chitosan-complexed dsRNA can be effectively delivered via feeding to *Bombyx mori* larvae, resulting in significant gene silencing and mortality [58]. This pioneering work suggests that oral administration of nanoparticle-formulated dsRNA is indeed a viable strategy for controlling lepidopteran insect pests. Therefore, further studies employing oral delivery of ds*MsCPs/SPc* to disrupt DDP represent a promising novel management strategy against *M. separata*.

## 5. Conclusions

In this study, we identified two CP-encoding genes that were highly expressed in gregarious larvae of *M. separata*. RNAi-mediated knockdown experiments provided strong evidence that upregulation of the two *MsCPs*-mediated changes in cuticle ultrastructure and thickness are associated with resistance to *B. bassiana* in gregarious *M. separata*. These novel findings broaden our understanding of insect DDP and are highly relevant in terms of successful biological control of *M. separata*.

## Data Availability

The original contributions presented in this study are included in the article/supplementary material. Further inquiries can be directed to the corresponding authors.

## Supplementary Materials

The following supporting information can be downloaded at: https://www.mdpi.com/article/10.3390/insects17040418/s1.
