# Supplementary material for: Upregulation of Two Cuticular Proteins Is Associated with Resistance to Beauveria bassiana in Crowded Mythimna separata
Source: Insects. 2026 Apr 15;17(4):418. doi: 10.3390/insects17040418 (PMC13117006; doi:10.3390/insects17040418)
Supplement: Supplementary file 1 [file insects-17-00418-s001.zip › Figure captions.pdf]

Figure S1. The nucleotide and deduced amino acid sequences of genes encoding cuticular proteins in *Mythimna separata*. Panels: *MsCP1* (A) and *MsCP2* (B). The start and stop codons are shaded in gray, and conserved chitin-binding domains are shown in blue font.

Figure S2. Phylogenetic analysis of 14 CPs in different insect species. Amino acids were aligned using ClustalW, and the tree was constructed with MEGA 7.0 and the neighbor-joining algorithm. Numbers at the nodes indicate percent bootstrap confidence values derived from 2,000 replications. The CPs analyzed were from the following species: GmCP1-like, *Galleria mellonella*; HkCP1-like, *Hyposmocoma kahamanoa*; HaCP1-like, *Helicoverpa armigera*; PiCP1-like, *Plodia interpunctella*; SlCP1-like, *Spodoptera litura*; VaCP1-like, *Vanessa atalanta*; AgCP1-like, *Aricia agestis*; SfCP1-like, *Spodoptera frugiperda*; TnCP1-like, *Trichoplusia ni*; SeCP1-like, *Spodoptera exigua*; MlCP1, *Mythimna loreyi*; MsCP1 and MsCP2, *Mythimna separata*.
