# Supplementary material for: Upregulation of Two Cuticular Proteins Is Associated with Resistance to Beauveria bassiana in Crowded Mythimna separata
Source: Insects. 2026 Apr 15;17(4):418. doi: 10.3390/insects17040418 (PMC13117006; doi:10.3390/insects17040418)
Supplement: Supplementary file 1 [file insects-17-00418-s001.zip › Figure S1.pdf]

A:CP1

```
1   ATGCAGTCGATCTGGTATCAGTTGCATAGTAGCCACCGAACAAAA
1     M  Q  S  I  W  Y  Q  L  H  S  S  H  R  T  K
46  CCTATCAAAATGAAATCCATGATCCTTGTCGCCCTCGCCCTCGTG
16    P  I  K  M  K  S  M  I  L  V  A  L  A  L  V
91  GCTGTCGCCGTGGCCGCCCCCGTGGAGGACTACACCCCCGTCGAA
31    A  V  A  V  A  A  P  V  E  D  Y  T  P  V  E
136 ATCGTCCGGTCCAAATTCGATTCGCAACCCGACGGCGCATACTCC
46    I  V  R  S  K  F  D  S  Q  P  D  G  A  Y  S
181 TTCGGATACGAAACCGCAGACGGCTCCGTCCGTGAAGAGACGGGT
61    F  G  Y  E  T  A  D  G  S  V  R  E  E  T  G
226 GAAGTGGTGGAAGCTCTGGATGAGGAGAACAAGCCCCACAACGTC
76    E  V  V  E  A  L  D  E  E  N  K  P  H  N  V
271 GTGGTCGTCAAAGGCTTCTACAGCTACGTCAACGCAGACGGTACC
91    V  V  V  K  G  F  Y  S  Y  V  N  A  D  G  T
316 CTCGAGACTATCAAGTACAAAGCTGACAGCCTAGGTTACAGCGCC
106   L  E  T  I  K  Y  K  A  D  S  L  G  Y  S  A
361 GAGGGACCCTCCATCCCTAAGGTCGAGAGCCAATAA
121   E  G  P  S  I  P  K  V  E  S  Q  *
```

B:CP2

```
1   ATGAAATCCATGATCCTTGTCGCCCTCGCCCTCGTGGCTGTC[illegible]
1     M  K  S  M  I  L  V  A  L  A  L  V  A  V
46  GTGGCCGCGCCCGTGGAGGACTACGTCGAAATCGTCCGGTCC[illegible]
16    V  A  A  P  V  E  D  Y  V  E  I  V  R  S
91  TACGATTCGAAACCCGACGGCGCATACAACTTCGGATATGAA[illegible]
31    Y  D  S  K  P  D  G  A  Y  N  F  G  Y  E
136 AGCGACGGCTCCACTCGTGAAGAGGCGGGTGAAGTGAAGAGT[illegible]
46    S  D  G  S  T  R  E  E  A  G  E  V  K  S
181 GTGGATGAGGAGAACAAGCCCCACGATGTCGTGGTCGTCCGC[illegible]
61    V  D  E  E  N  K  P  H  D  V  V  V  V  R
226 TCCTTCTCCTACGTCAACCCTGATGGTGTAACCGAAACTATC[illegible]
76    S  F  S  Y  V  N  P  D  G  V  T  E  T  I
271 TACTACGCTGACGAGAACGGTTACCATGCCGAGGGACCTTCC[illegible]
91    Y  Y  A  D  E  N  G  Y  H  A  E  G  P  S
316 CCCAAGGCCGTGAGGCGATAA
106   P  K  A  V  R  R  *
```
