# Supplementary material for: Upregulation of Two Cuticular Proteins Is Associated with Resistance to Beauveria bassiana in Crowded Mythimna separata
Source: Insects. 2026 Apr 15;17(4):418. doi: 10.3390/insects17040418 (PMC13117006; doi:10.3390/insects17040418)
Supplement: Supplementary file 1 [file insects-17-00418-s001.zip › Table S1.pdf]

Table S1 Primers used in this study

Primer name	Sequence (5'-3')	Size（bp)	Usage
qCP1-F	GAAGAGACGGGTGAAGTGGTG	80	qRT-PCR
qCP1-R	CTGTAGAAGCCTTTGACGACCA		
qCP2-F	GGAGAACAAGCCCCACGATG	128	
qCP2-R	AAGGTCCCTCGGCATGGTAA		
Actin-F	AACTTCCCGACGGTCAAGTCAT	168	
Actin-R	TGTTGGCGTACAAGTCCTTACG		
Tubulin-F	CGGTAATGCCTGCTGGGAA	118	
Tubulin-R	CTCGCTGAAGAAGGTGTTGAA		
dsCP1-F	taatacgactcactatagggTGCATAGTAGCCACCGAACA	212	dsRNA synthesis
dsCP1-R	taatacgactcactatagggCACCACTTCACCCGTCTCTT		
dsCP2-F	taatacgactcactatagggAAATCCATGATCCTTGTCGC	176	
dsCP2-R	taatacgactcactatagggACACTCTTCACTTCACCCGC		
