# Supplementary figures and images for: Upregulation of Two Cuticular Proteins Is Associated with Resistance to Beauveria bassiana in Crowded Mythimna separata
Source: Insects. 2026 Apr 15;17(4):418. doi: 10.3390/insects17040418 (PMC13117006; doi:10.3390/insects17040418)

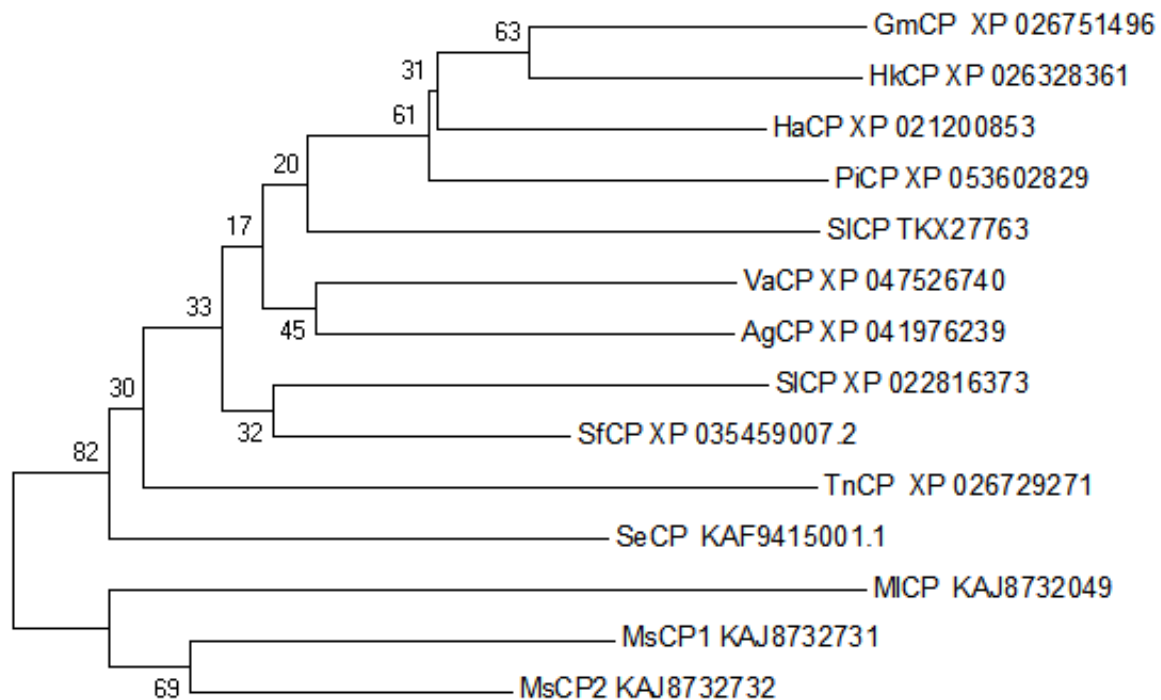

0.050

Supplement: Supplementary file 1 [file insects-17-00418-s001.zip › Figure S2.pdf]
